# Extension of the Shelf Life of Sliced Sponge Cake Through a Combination of Modified Atmosphere and Active Packaging with Hydroxytyrosol and Eugenol

**DOI:** 10.3390/foods14234093

**Published:** 2025-11-28

**Authors:** Djamel Djenane, Mohammed Said Metahri, Mohammed Aider, Agustín Ariño, Nuria López Aznar

**Affiliations:** 1 Meat Quality and Meat Safety Research Group, University Mouloud Mammeri, Tizi Ouzou 15000, Algeria; 2 Facultad de Veterinaria, Instituto Agroalimentario de Aragón-IA2, Universidad de Zaragoza-CITA, 50013 Zaragoza, Spain; aarino@unizar.es; 3 Faculty of Biological and Agricultural Sciences, Mouloud Mammeri University, Tizi Ouzou 15000, Algeria; metahriummtodz@gmail.com; 4 Department of Soil Sciences and Agri-Food Engineering, Université Laval, Quebec City, QC G1V 0A6, Canada; mohammed.aider@fsaa.ulaval.ca; 5 Institute of Nutrition and Functional Foods (INAF), Université Laval, Quebec City, QC G1V 0A6, Canada; 6 AIMPLAS–Technological Institute of Plastics, València Parc Tecnològic C/Gustave Eiffel 4, 46980 Paterna, Spain; nlopez@aimplas.es

**Keywords:** sponge cake, modified atmosphere packaging, bioactive films, storage, shelf life

## Abstract

The development of bioactive food packaging is an important issue, given its potential to preserve food quality and safety without the use of synthetic preservatives. This study aimed to develop new polystyrene foam (PS) films with hydroxytyrosol (HOxTYR) and eugenol (EUG), alone or in combination, as bioactive molecules to preserve sliced sponge cake during long-term storage. The cake samples were analyzed periodically during storage at 15 °C in terms of quality attributes (pH, water activity, height, volume and weight loss, firmness, CIE Lab color, lipid peroxidation products, microbial spoilage, and overall acceptability) and shelf life. The active film containing the combination of 0.6% HOxTYR and 0.6% EUG showed the strongest antioxidant activity, which was attributed to a potential synergism between the compounds, resulting in lower lipid oxidation rates (TBARS). The combination of HOxTYR and EUG also offered the greatest reduction in bacterial load (62% for *S. aureus* and 58% for *E. coli*), suggesting a synergistic effect on microbial inhibition. Likewise, samples packaged in a modified atmosphere (MAP) with the active film containing the combination of HOxTYR and EUG showed the best performance, including a smoother texture and greater volume, more stable color, lower microbial counts, and greater overall acceptability, and, consequently, a longer shelf life of up to 70 days at room temperature. Furthermore, the results of this study could contribute to environmental protection by reducing food waste, and suggest that the developed active packaging technique represents a promising and innovative approach to the preservation of bakery products.

## 1. Introduction

Globally, bread and bakery products are among the most widely consumed goods, and their supply chain has recently experienced significant growth. The global bakery products market was valued at USD 248.8 billion in 2024 and is estimated to grow at a compound annual growth rate (CAGR) of over 4.4% from 2025 to 2034, potentially reaching USD 394.3 billion [1]. Within the bakery industry, sponge cake (SC) is a widely consumed product worldwide, valued for its characteristic flavor, attractive color, and texture. Sponge cakes are considered ready-to-eat (RTE) foods as they require no preparation before consumption.

The edibility of sponge cake during storage can be affected by intrinsic factors such as water activity, moisture, pH value, and microbial load, as well as extrinsic factors like relative humidity, temperature, gas composition, light exposure intensity, and packaging material [2,3]. A serious and costly issue for the bakery industry is the economic loss caused by physicochemical and microbial spoilage, which often represents the main factor limiting the shelf life of intermediate and high-moisture bakery goods [4,5]. Most commercially available cakes are conventionally packaged in air and displayed on shelves at room temperature rather than being refrigerated. Unfortunately, some RTE products have been associated with outbreaks of foodborne illness globally [6]. RTE foods, like all foods, can become contaminated during production, handling, packaging, and distribution. Furthermore, because these foods are consumed ‘as is’, the presence of pathogenic bacteria represents an increased risk of foodborne illness.

Various synthetic food preservatives are used to inhibit microbial growth and extend the shelf life of bakery products. Common chemical preservatives include propionates, sorbates, benzoates, and parabens [7]. These compounds are often used in combination with other additives to achieve optimal preservation. However, these preservatives can negatively affect product quality, causing undesirable changes, such as imparting perceptible off-flavors (e.g., bitter notes from propionates at higher concentrations) or altering the functionality of key ingredients, which can compromise the product’s sensory profile and texture. Moreover, their use conflicts with the growing consumer demand for clean-label food products, prompting the need for natural alternatives, such as plant-based substances [8,9,10].

Long-distance transport and subsequent retail display of manufactured sponge cake can negatively affect water loss, texture, microbial load, and appearance. To extend shelf life and maintain quality, packaging is prioritized, leading to the consideration of alternatives such as modified atmosphere packaging (MAP) or active packaging [11,12,13]. MAP provides a useful method for extending the shelf life of cakes by restricting the growth of harmful microorganisms. This involves using high-barrier polymeric packaging with elevated carbon dioxide (CO_2_) and balanced nitrogen (N_2_) for bacteriostatic and fungistatic activities. Oxygen (O_2_) must be reduced to the critical level necessary to suppress the activity of aerobic microorganisms, requiring the elimination of O_2_ entry through the packaging barriers during storage. However, insufficient research has been conducted on the effects of MAP on the quality and microbial characteristics of stored bakery goods such as sponge cakes.

The packaging sector is one of the largest in the global plastics industry, having experienced continuous growth in recent years due to the diversity of its products and applications [14]. Its development over the last two decades makes it a viable alternative for reducing the environmental impact of conventional plastics. One of the most common strategies for extending the shelf life of food products is the incorporation of antioxidant and antimicrobial compounds into food packaging materials [15,16,17,18]. Furthermore, improving the quality and extending the shelf life of food while maintaining its organoleptic and nutritional properties is generating great interest in the scientific and industrial fields. The main focus of developing these materials has been studying their functional properties by using methods to determine their antimicrobial and antioxidant behavior, as well as their disintegration, toxicological, and migration properties.

Due to their antimicrobial and antioxidant characteristics and natural origin [19,20,21], hydroxytyrosol (3,4-dihydroxyphenylethanol) (HOxTYR) and eugenol (4-Allyl-2-methoxyphenol) (EUG) were selected in our study for addition to the new active formulations based on polystyrene (PS). Because of these properties, they are being investigated for use in active packaging, where materials interact with food or the environment to extend shelf life, preserve quality, and enhance food safety. Both hydroxytyrosol and eugenol have GRAS (Generally Recognized as Safe) status for use in foods [22,23]. Another important feature of HOxTYR and EUG in food packaging systems is their stability and the possibility of controlled release over time [24,25]. In fact, the release rate is a key parameter for enabling adequate and sustained microbial inhibition and antioxidant activity of both biomolecules [26].

To the best of our knowledge, there are no reported studies regarding the application of PS foamed films enriched with HOxTYR and EUG as active packaging combined with modified atmosphere packaging in the preservation of soft bakery products. Therefore, the primary aim of this study was to develop and evaluate a novel active packaging system for the preservation of sliced sponge cake (SC). This involved three specific objectives:

Film Development: To incorporate two potent natural bioactive compounds, hydroxytyrosol (HOxTYR) and eugenol (EUG), individually and in combination, into polystyrene foam (PS) films to create new active packaging materials.

Efficacy Assessment: To investigate the synergistic effect of combining the developed active film with modified atmosphere packaging (MAP: 40% CO_2_/60% N_2_) on the quality preservation of sliced sponge cake during a long-term storage study (70 days) at 15 °C.

Quality Evaluation: To comprehensively monitor and compare the effect of the different packaging strategies (Air, MAP, and active films) on critical quality attributes, including lipid oxidation (TBARS), microbial stability, physicochemical properties (pH, firmness, color), and overall shelf-life extension and safety of the sponge cake.

## 2. Materials and Methods

### 2.1. Raw Materials and Chemicals

The raw materials used for the sponge cake production were: wheat flour (Groupe Labelle, Spa, El-Harrach district, Algiers province, Algeria), powdered white fine sugar (CevitaL, Spa, Béjaia, Algeria), pasteurized liquid whole eggs (OVO GIDKY^®^ Sarl, Naciria, Algeria), low-fat powder milk (Almag, Akbou, Algeria), soybean oil (CevitaL, Spa, Béjaia, Algeria), baking powder (Noura, Tizi Rached, Algeria), vanilla (Safpal Idéal, Oran, Algeria), and locally purchased salt.

**Figure 1:**
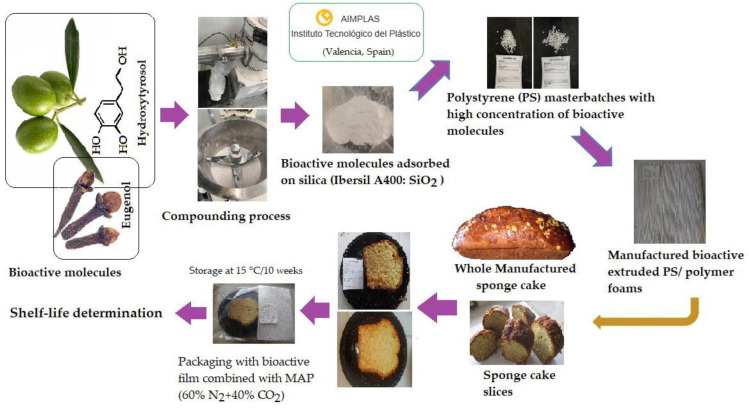
Conceptual diagram of the methodology followed in the present study.

For the active films, commercial polystyrene (PS), PS Crystal 1160 (TotalEnergies Corbion, Gorinchem, The Netherlands), and silica Ibersil A400 (Industrias Químicas del Ebro, S.A., Zaragoza, Spain) were used. Hydroxytyrosol (HOxTYR) (Econatur, La Carlota, Córdoba, Spain) and eugenol (EUG) (Cymit Química S.L., Barcelona, Spain) served as the bioactive agents. A conceptual diagram outlining the methodology followed in this study is shown in Figure 1.

### 2.2. Development of PS-Based Hydroxytyrosol and Eugenol Bioactive Polymers

**Figure 2:**
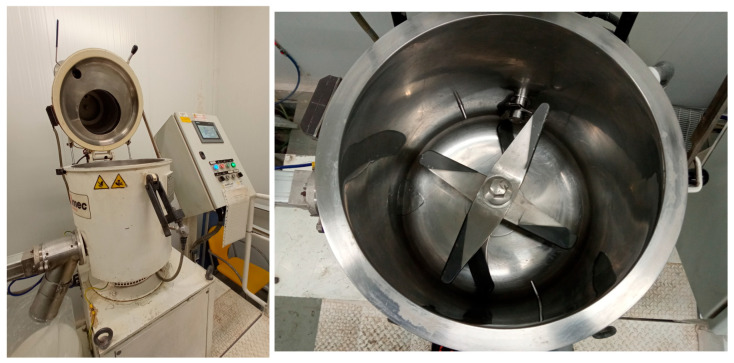
Turbo-mixer for the adsorption process of active compounds onto silica.

The new bioactive films were produced at the AIMPLAS Plastics Technological Institute (Paterna, Valencia, Spain). To facilitate their incorporation into the extruder and control the subsequent release of the active ingredients, the selected natural bioactive compounds (HOxTYR and EUG) were first adsorbed onto an inorganic payload (inert silica Ibersil A400). To achieve the correct adsorption of the active compounds on silica, the ingredients were mixed in a 10-litre turbo-mixer as shown in Figure 2.

As a result, the dry mixtures were obtained in powder form, which was used for dosing in the formulation process. From the selected polymers and the dry mixtures produced, a series of compounds was obtained using a co-rotating twin-screw extruder (Coperion ZSK 25), with an aspect ratio L/D = 40, a diameter D = 25, and 6 length modules (from Zone 0 to Zone 5). The materials were fed into the extruder using different gravimetric feeders, which can control the material flow (kg/h) of each component of the formulation with high precision. The polymer (PS) was fed through Zone 0 of the extruder, and the dry mixtures through Zone 2 of the extruder.

The temperature profiles used across the extruder’s heating zones (from Zone 0 to Zone 5) were 190/185/180/175/175/170 °C. The key characteristics of the polystyrene (PS) foam films are primarily related to their excellent thermal properties, light weight, and rigidity, but they are generally known for poor gas barrier properties and low resistance to water vapor. That is why the research on active sponge cake packaging used a PS foam film as a carrier for active compounds, but it relies on a separate, high-barrier material (polyamide/polyethylene PA/PE plastic bag) to achieve the modified atmosphere required for long-term preservation. The PS films were manufactured in thin gauges of about 75 microns (0.075 mm) with a low density of approximately 1 g/cm^3^ for the solid polymer.

**Figure 3:**
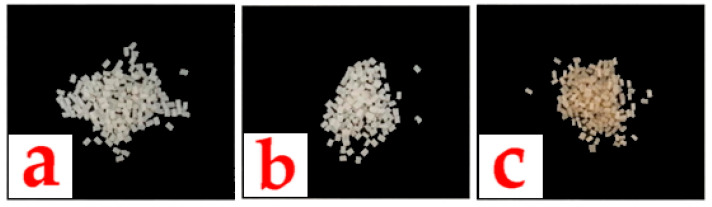
Pellet granules of (**a**) PS/neat polystyrene, (**b**) PS/EUG with eugenol, and (**c**) PS/HOxTYR with hydroxytyrosol. Note the light yellow-to-brownish hue of pellets in sample c, confirming the successful incorporation of the naturally colored HOxTYR.

According to the processing conditions, a screw configuration was designed to provide shear to the materials and achieve proper mixing within the extruder between the polymer matrix and the dry blend (inert silica with bioactive compounds). The concentrations of bioactive compounds ranged from 0.6 to 1%, which is the optimal amount for the sponge cake obtained in our study through preliminary tests. The strands coming out of the extruder nozzle were continuously cooled in a water bath and cut into 3 mm long granules, as shown in Figure 3.

This research focuses on new masterbatch compositions with a high concentration of the bioactive compounds hydroxytyrosol and eugenol, either alone or in combination. It also focuses on a process for the production of this new composition and its use in the production of bioactive polymers for food packaging. In the present study, the foamed PS films obtained were a control without bioactive compounds and three test PS films containing HOxTYR (1%), EUG (1%), and a combination of HOxTYR (0.6%) and EUG (0.6%). The changes in mechanical properties due to the incorporation of HOxTYR and EUG were minor and the film retained sufficient structural integrity for the intended use in packaging. The active films with HOxTYR showed a slight but acceptable increase in the b* (yellowness) value, confirming the incorporation and slight coloration of the natural compound, but maintaining overall visual acceptability.

### 2.3. In Vitro Antioxidant and Antimicrobial Activity of the PS-Based Bioactive Films

#### 2.3.1. DPPH Radical-Scavenging Activity

The antioxidant activity of the active PS foamed films with HOxTYR and EUG was determined using the DPPH assay. At first, film samples (1.5 × 1.5 cm^2^) were mixed with 10 mL of ethanol for 15 min at room temperature away from light. Then, the supernatant was separated from the decanted part and centrifuged for 10 min at high speed. 100 μL of film extract was mixed with 2 mL of the 40 ppm (0.004%) DPPH ethanol solution in a test tube. After 30 min in the dark, the absorbance (A) was determined at 517 nm using a spectrophotometer (Shimadzu, Tokyo, Japan). The radical-scavenging activity (RSA) was calculated according to Equation (1):

(1) $$RSA\left(\%\right)=\frac{A\left(DPPH\right)-A\left(Bioactive\;compound\right)}{A\left(DPPH\right)}\times 100$$

The antioxidant activity was expressed as IC_50_ value (μg/mL), the concentration required to cause 50% of DPPH inhibition. Synthetic antioxidant reagent butylated hydroxytoluene (BHT) (Fluka, Sigma-Aldrich Chemie GmbH, Barcelona, Spain) was used as a positive control. To standardize DPPH results, the antioxidant activity index (AAI) proposed by Scherer and Godoy [27] was calculated as indicated in Equation (2):

(2) $$AAI=\frac{\left[DPPH\right]\;in\;reaction\;mixture\left(µg/mL\right)}{{IC}_{50}\left(µg/mL\right)}$$

The antioxidant activity is considered poor when AAI < 0.5, moderate when AAI is between 0.5 and 1.0, strong when AAI is between 1.0 and 2.0, and very strong when AAI > 2.0.

#### 2.3.2. Antimicrobial Activity

The antimicrobial activity of the control (neat PS) and the active PS foamed films with HOxTYR and EUG was determined against *Staphylococcus aureus* CECT 4459 (*S. aureus*, Gram-positive) and *Escherichia coli* O157:H7 CECT 4267 (*E. coli*, Gram-negative). The foodborne pathogens used in this work were purchased from the Collection Española de Cultivos Tipo (CECT) and were grown on tryptic soy agar (TSA) and MacConkey plates, respectively, and incubated at 37 °C for 24 h. Cultures were grown in tryptic soy broth (TSB) at 37 °C until reaching a bacterial load of 1 × 10^7^ CFU/mL for *S. aureus* and *E. coli*. The bacterial inoculum (0.1 mL) was distributed evenly over the test films (3.0 × 3.0 cm^2^) using sterile disposable cell spreaders. To prevent evaporation, the test films were placed in sterile glass Petri dishes sealed with a sterile sample bag. Samples were incubated at 37 °C for 24 h. After incubation, each film sample was rinsed thoroughly with 10 mL of sterile phosphate-buffered saline solution. The bacterial suspensions were subjected to serial dilution and plated agar onto nutrient agar incubated at 37 °C for 48 h. The antimicrobial activity (AM) was calculated according to Equation (3):

(3) $$AM\left(\%\right)=\frac{A-B}{A}\times 100$$

where A is the bacterial count (CFU/mL) for the control (5 log CFU/mL) and B is the CFU/mL for the test films with hydroxytyrosol (PS/HOxTYR), eugenol (PS/EUG) and both bioactive compounds (PS/HOxTYR + EUG), respectively.

### 2.4. Sponge Cake Preparation and Packaging

#### 2.4.1. Sponge Cake Preparation and Composition

The sponge cake (SC) was prepared according to the standardized industrial recipe using the following ingredients (based on the total weight of the cake formulation): wheat flour (32.15%), refined sugar (22.50%), pasteurized liquid whole eggs (19.29%), soybean oil (9.64%), powder milk (1.60%), salt (0.64%), vanilla (0.32%) and water (12.86%). The cake batter was baked for 20 min at 200 °C in a preheated electric oven for 15 min (Panasonic, Osaka, Japan). After baking, the whole cakes have been unmolded and left to cool for an hour at room temperature before being placed in sealed plastic bags to prevent them from drying out.

Methods provided by the Association of Official Analytical Chemists [28] were used to determine the chemical composition of sponge cake. Moisture content was determined by drying at 130 °C in a controlled oven. The Soxhlet extraction method was employed to determine the level of crude fat using petroleum ether as solvent. Crude protein content was determined by the Kjeldahl method using a conversion factor of 6.25. Ash content was obtained after incineration at 600 °C for 3 h in a muffle furnace. Total carbohydrates were calculated by difference. Finally, energy values for 100 g (kJ and kcal) were calculated using the conversion factors specified in Regulation (EU) No 1169/2011 [29] (4 kcal/g and 17 kJ/g for protein and carbohydrates, 9 kcal/g and 37 kJ/g for fat). The proximate composition of the freshly manufactured sponge cakes used in this study was (values per 100 g): moisture 16%, protein 9%, fat 19%, carbohydrates 56%, ash 1%, energy 1791 kJ/427 kcal.

#### 2.4.2. Sponge Cake Packaging

The sponge cake (SC) was aseptically cut into portions (99.50–120 g each) that were randomly divided into five groups: (a) SC-0 control packaged in air, (b) SC-1 control packaged in MAP, (c) SC-2 packaged in MAP with film containing 1% hydroxytyrosol (d) SC-3 packaged in MAP with film containing 1% eugenol, and (e) SC-4 packaged in MAP with film containing 0.6% hydroxytyrosol plus 0.6% eugenol (Table 1).

**Figure 4:**
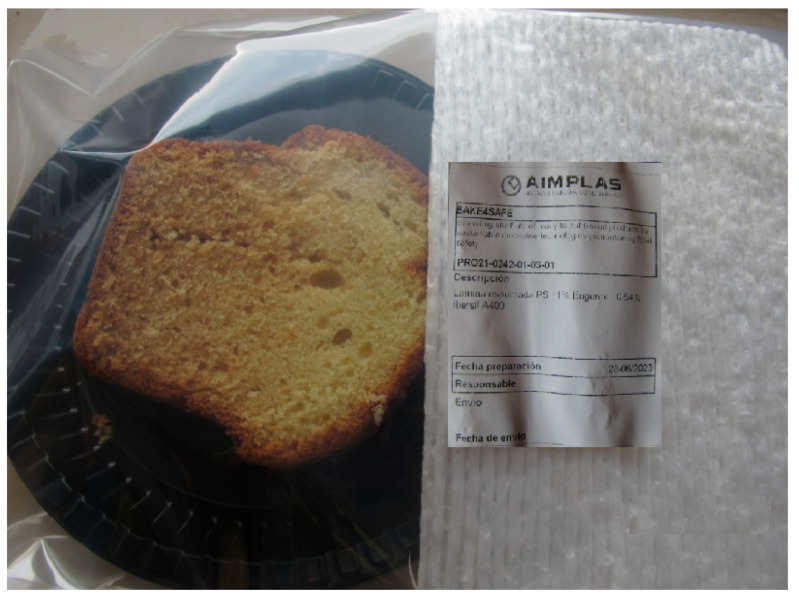
Appearance of a slice of cake wrapped in one of the films studied.

The SC portions were placed individually in a polystyrene tray and wrapped in a polyamide/polyethylene (PA/PE) plastic bag (Irma, Zaragoza, Spain), which acted as a barrier to create a closed system. Subsequently, the bioactive PS films were placed covering 50% of the upper inner surface, leaving a headspace between the film and the SC portion (Figure 4). The use of a partial active film covering only 50% of the internal surface of food packaging was primarily based on a balance between effectiveness and practical considerations such as cost efficiency and targeted protection. However, the analysis of the release kinetics data was beyond the scope of this initial study, which primarily focused on the immediate efficacy (e.g., in terms of radical scavenging and immediate preservation) and the synergistic effect of the compounds incorporated into the film. The lack of a study of the release kinetics of the active compounds is a limitation of the present research.

All trays (except control 1) were filled with a gas mixture of 40% CO_2_ + 60% N_2_. The volume injected into the headspace was approximately 2 L in a ratio product/gas mixture 1:2 (*v*/*v*). All samples were stored in obscurity at 15 °C and a relative humidity (RH) of 65% for 10 weeks. Samples for physical-chemical, microbiological and sensorial analysis were taken at day 0 and then after 10, 20, 30, 40, 50, 60 and 70 days of storage.

The headspace atmosphere composition in each package of the SC sample was monitored using a Hewlett Packard 4890 gas chromatograph equipped with a thermal conductivity detector. Samples of 50 µL were injected into a Chromakey CP-carboplot P7 column of 0.53 mm inner diameter and 27.5 m length, with helium as the carrier gas at a flow rate of 12.6 mL/min. The initial temperature of the oven was set at 40 °C. After 2.5 min, the oven temperature was raised at a rate of 45 °C/min to a final temperature of 115 °C. The temperature of the injector block was 59 °C. The temperature of the detector was 120 °C. A calibration curve was prepared using a 40% CO_2_/60% N_2_ atmosphere (Abelló Linde S.A., Barcelona, Spain). The total percentage of each gas was calculated from the average of three measurements of the appropriate peak, and the results were expressed as %CO_2_ and %N_2_.

### 2.5. Determination of pH and Water Activity of Sponge Cake

The pH of SC samples was measured using a micro pH-meter model 2001 (Crison Instruments, Barcelona, Spain) after homogenizing 3 g of sample in 27 mL distilled water for 10 s at 1300 rpm with an Ultra-Turrax. The mixture was then filtered for pH determination. Water activity (a_W_) was measured with an accuracy of ± 0.003 in a dew point hygrometer aWLife (Scharlab S.L., Barcelona, Spain) (AquaLab 4TE). Each value of pH and a_W_ was the mean of three replicates.

### 2.6. Changes in Weight, Height, Volume and Texture of Sponge Cake

The initial and the final weight of each packaged SC was measured using an electronic precision balance (Ohaus^®^, Merck KGaA, Darmstadt, Germany: 0.001 g) after opening the package at room temperature for 15 min. The calculations took into account that the sampling of cake slices generated portions of different sizes depending on their previous asymmetrical condition and, therefore, their different heights and weights.

Weight loss was calculated according to the following Equation (4):

(4) $$WL\left(\%\right)=\frac{W_{0}-W_{s}}{W_{0}}\times 100$$

where WL is the weight loss (%), W_0_ is the initial weight (g) on day 0, and W_s_ is the measured weight (g) of each sample at each sampling point.

The height (cm) of the SC was measured using a digital caliper (Z136115-1EA, Merck Life Science S.L.U., Madrid, Spain). Five measurements were taken from different sides of the SC and the average of the five points was recorded. The volume (cm^3^) of the sponge cake was measured using the rapeseed displacement method No. 10-05-01 [30].

For cake firmness, the hardness parameters of the Texture Profile Analysis (TPA) curve were considered. The maximum force required to penetrate an SC was measured with a Texture Analyzer (TA-XT2i, Texture Technologies, Hamilton, MA, USA) with a 100 mm diameter cylindrical probe. The test was performed on the cubic pieces of the sponge cakes (30 × 30 × 30 mm^3^) after removing of the crust. At least five slices of randomly selected sponge cake of similar size were measured per sampling point and per package of approximately 3 × 3 × 3 cm^3^ (height × width × length). A test speed of 2 mm/second and a distance of 15 mm were used. The sample was compressed to 50% of its original height with a duration of 30 s of evaluation. Firmness was expressed as the maximum penetration force (N), while the extension at maximum penetration force was also recorded. All measurements were carried out in a controlled room at 20–25 °C, 15 min after opening the packaging. Penetration force data from five slices were recorded, and the average of five readings was used for analysis. Results were expressed as load in grams.

### 2.7. Crumb Color Measurement of Sponge Cake

The color of the SC crumb was evaluated by measuring the L* (lightness), a* (redness), and b* (yellowness) parameters (CIELAB color space) using a color difference meter (CR-400, Minolta Co. Ltd. Osaka, Japan). Before the measurements, the colorimeter was calibrated with a white reference plate provided with the instrument. To determine the color difference between the control SC and the SC packed with active films, the total color difference (ΔE*) was calculated by comparing the difference between the control and treated sample for each component (ΔL*, Δa* and Δb*). A low ΔE* value indicates a small color difference, meaning the colors are very similar. Generally, a difference of less than 1.00 is not perceptible to the human eye, while a value close to 3.00 is considered a significant difference, meaning the color difference is evident to the human eye.

### 2.8. Measurement of Lipid Peroxidation of Sponge Cake

Lipid peroxidation was measured by thiobarbituric acid reaction substances (TBA-RS) according to a previously published method with minor modifications [19]. Briefly, crumb SC samples (10 g) were mixed with 20 mL of 10% trichloroacetic acid and centrifuged in tubes at 2300 g for 30 min at 4 °C, and the supernatants were filtered through MN 640W filter paper (Machinery-Nagel GmbH & Co. KG, Düren, Germany). The collected supernatant (500 µL) was mixed with an equal volume (500 µL) of 20 mM thiobarbituric acid and vigorously vortexed. After that, the test tubes were placed in a boiling water bath for 20 min and cooled. After cooling, the absorbance was measured at 532 nm using a spectrophotometer. TBARS values were calculated from a standard curve of malonaldehyde and expressed as mg malonaldehyde/kg of the sponge cake.

### 2.9. Microbiological Analysis of Sponge Cake

SC samples (10 g) were homogenized in 90 mL of peptone water (0.1%) with salt (0.85%) using a stomacher for 2 min and a series of decimal dilutions was carried out. In order to determine total aerobic mesophilic bacteria, yeast, and mold counts, 1 mL of serially diluted samples was pour-plated on plate count agar (PCA) and potato dextrose agar with chloramphenicol (PDAC) plates, respectively. Total viable count plates were incubated aerobically for 48 h at 37 °C, while yeast and mold plates were incubated for 5 days at 25 °C. Colonies were counted and results expressed as log CFU (colony-forming units)/g of sample.

### 2.10. Sensory Evaluation of Sponge Cake

Hedonic test was used to determine the degree of overall acceptability scores for sponge cakes according to the AACC method 10–90 with modifications. For this study, 30 sensory panelists were recruited from the Mouloud MAMMERI University (Algeria). Three portions of each SC sample were presented in random order and were evaluated for overall acceptability. Participants were asked to evaluate acceptability levels for sponge cakes using a 7-point hedonic scale (7 = like extremely, 6 = like moderately, 5 = like slightly, 4 = neither like nor dislike, 3 = dislike slightly, 2 = dislike moderately, and 1 = dislike extremely). The samples were then placed on plates and assigned random three-digit numbers for identification purposes.

### 2.11. Statistical Analysis

All analyses (physicochemical, chemical, and microbiological) were performed in triplicate (*n* = 3) on independent batches of packaged sponge cake. Statistical analysis was performed with an SPSS computer package Version 16.0 (SPSS Inc. Chicago, IL, USA). The analysis of variance (ANOVA) was performed to evaluate the effect of packaging type, storage time, and their interactions on the quality parameters and microbial levels. Tukey’s multiple range tests were used for a mean comparison at the 95% significance level. The necessary assumptions for the application of ANOVA and Tukey’s tests were previously analyzed by the Shapiro–Wilk test to check for the normality of data, and the Levene’s test for the homogeneity of variances.

## 3. Results and Discussion

### 3.1. Antioxidant Activity of the PS-Based Bioactive Films

Foamed polystyrene (PS) does not inherently possess significant antioxidant properties but can be functionally enhanced by incorporating antioxidant compounds into the polymer. The results of the in vitro antioxidant capacity of the PS-based polymer confirmed that the addition of bioactive molecules such as hydroxytyrosol (HOxTYR) and eugenol (EUG) successfully conferred antioxidant properties to the films (Table 2).

According to our results, the inhibition of the DPPH radical increased significantly when the two molecules were combined. This enhanced effect is not merely additive but is attributed to a chemical synergy between hydroxytyrosol and eugenol that improves the overall radical-scavenging efficiency. Specifically, the combination allows HOxTYR to be regenerated after it has quenched a radical. As a powerful primary antioxidant, HOxTYR quenches the DPPH radical by donating a hydrogen atom. Eugenol, acting as a secondary antioxidant, can then potentially donate a proton or electron to the oxidized HOxTYR radical, effectively restoring it to its original, active HOxTYR form. This regeneration process allows the combined system to scavenge more radicals than the simple sum of the individual contributions, resulting in the observed higher DPPH inhibition.

The IC_50_ values for the PS/HOxTYR and PS/EUG films were 49.25 µg/mL and 69.28 µg/mL, respectively. In contrast, the IC_50_ value for the combination (PS/HOxTYR + EUG) was 29.43 µg/mL, which is 1.67 and 2.35 times lower (*p* < 0.05) than the individual films, indicating a significantly stronger antioxidant activity. For comparison, the positive control, butyl hydroxytoluene (BHT), obtained an IC_50_ value of 15.3 ± 0.1 µg/mL.

A significant amount of the phenol groups in HOxTYR and EUG with DPPH radical scavenging capacity remained active after polymer manufacturing and processing. Consequently, these polymers enriched with both bioactive molecules showed the promised functional benefits. The DPPH inhibition values could be correlated with the amount of antioxidant molecules released by the polymers. Our findings are similar to those previously reported by Luzi et al. [31], who examined hydroxytyrosol-enriched extracts as active antioxidant ingredients for poly(vinyl alcohol) (PVOH) based films, also using the DPPH assay.

According to the Antioxidant Activity Index (AAI), the PS/HOxTYR + EUG polymer exhibited strong antioxidant activity (AAI = 1.37), likely due to the synergism between the two bioactive compounds. Both HOxTYR and EUG are known for their antioxidant properties, which consequently delay lipid oxidation in foods. Gavaric et al. [32] found a comparable synergistic effect when combining carvacrol and thymol at 4% by weight, equivalent to the activity observed when both compounds were used separately at 8%, as measured by the DPPH test.

The synergistic effect between hydroxytyrosol (HOxTYR) and eugenol (EUG) primarily stems from their complementary antioxidant mechanisms. Both are phenolic compounds, but their chemical structures offer different ways to scavenge radicals, resulting in a more effective combined action. HOxTYR is a potent, hydrophilic antioxidant due to its catechol moiety (the o-dihydroxyphenyl group). Its primary actions are hydrogen atom transfer, where it rapidly donates a hydrogen atom to free radicals to break the radical chain reaction, and metal chelation, preventing radical formation by binding to metal ions (like Fe or Cu) that catalyze lipid oxidation.

EUG is a constituent of essential oils and is generally more lipophilic than HOxTYR. It also primarily acts as a chain-breaking antioxidant through hydrogen atom transfer via its single hydroxyl (OH) group. In the presence of another scavenger like HOxTYR, eugenol can act as a secondary antioxidant, contributing to the overall free radical scavenging capacity. The simultaneous scavenging of radicals by both HOxTYR and EUG leads to a greater-than-additive (synergistic) effect. Additionally, synergy in radical quenching may be explained by radical regeneration, a key mechanism in antioxidant mixtures. It is hypothesized that the more potent scavenger (HOxTYR) neutralizes a primary radical, and the resulting HOxTYR-radical is then reduced back to its active form by the second compound (EUG), which is often consumed in the process. This regeneration effectively allows the more potent antioxidant to function for a longer period or in a higher capacity before being irreversibly consumed.

Our results suggest that a significant amount of hydroxytyrosol and eugenol remained intact in the polymer matrix after processing and could consequently act as active agents in PS-based formulations. Therefore, the PS-based bioactive polymers obtained in this study could be used as antioxidant films for food packaging to extend the shelf life of food products by slowing the oxidation process.

The antioxidant capacity of the developed films was evaluated using the 2,2-diphenyl-1-picrylhydrazyl (DPPH) radical scavenging assay, which quantifies the capacity of the active films to prevent lipid oxidation, a primary spoilage pathway in high-fat bakery products like sponge cake. The highest DPPH inhibition was achieved by the film containing the combination of 0.6% HOxTYR and 0.6% EUG, significantly surpassing the activity observed for the individual HOxTYR and EUG films. Results showed the formation of an effective, functional packaging material where the combined compounds exert a synergistic effect, maximizing the scavenging of free radicals and supporting the rationale for their use in food preservation [33,34].

HOxTYR, primarily found in olives and olive oil, is a potent antioxidant that can scavenge free radicals, reduce oxidative stress, and protect against cellular damage [35,36]. EUG, found in cloves and other spices, also exhibits antioxidant activity by scavenging free radicals and preventing the formation of reactive oxygen species [37]. Adding antioxidants such as plant polyphenols could slow the accumulation of free radicals, thereby increasing oxidative stability and reducing the accumulation of secondary oxidative substances in stored food matrices [38,39]. For example, Aguado et al. [34] found that packaging paper coated with olive extract rich in hydroxytyrosol successfully inhibited oxidative reactions. Orlo et al. [33] found that the DPPH-scavenging activity significantly improved with coated eugenol. Arumsari et al. [40] found that a biodegradable active film containing ascorbyl palmitate and sodium ascorbyl phosphate as antioxidants effectively enhances antioxidant capacity and reduces UV transmittance. These properties make the films suitable for use in biodegradable, antioxidant-enriched packaging solutions for preserving foods prone to oxidative degradation.

### 3.2. Antimicrobial Activity of the PS-Based Bioactive Films

The active PS film containing hydroxytyrosol (HOxTYR) reduced the bacterial load by only 8% for *S. aureus* and 4% for *E. coli*. In contrast, the active PS film containing eugenol (EUG) reduced the bacterial load by 42% for *S. aureus* and 36% for *E. coli*, respectively. However, the film manufactured with a combination of both bioactive compounds showed significant reductions of 62% for *S. aureus* and 58% for *E. coli* (Table 3). This superior performance may be due to the synergistic effects of the two phenolic compounds, which work together to enhance bacterial inhibition.

Eugenol, a major component of clove essential oil, is widely recognized as a broad-spectrum antimicrobial agent, highly effective against bacteria, yeast, and mold. Eugenol’s lipophilic nature allows it to disrupt the microbial cell membrane. It integrates into the cell wall and mitochondrial membranes, increasing permeability, which leads to the leakage of intracellular components (e.g., ATP, proteins) and ultimately causes cell death.

While hydroxytyrosol is primarily known for its potent antioxidant activity, it also possesses moderate antimicrobial properties, especially against certain foodborne pathogens and spoilage microorganisms. Hydroxytyrosol’s action is mainly due to its catechol structure (two adjacent hydroxyl groups on the aromatic ring). This structure allows it to interfere with bacterial metabolism, potentially by chelating metal ions essential for microbial enzyme activity, or by generating reactive oxygen species in the cell, leading to oxidative damage.

The superior antimicrobial performance of the combined PS/HOxTYR + EUG film (resulting in a 62% reduction in *S. aureus* and 58% in *E. coli*) is attributed to a potential synergistic effect. While EUG rapidly compromises the microbial cell membrane via volatilization, HOxTYR, through slow migration by diffusion, causes metabolic interference and metal chelation. This dual action provides a sustained secondary attack, making the cells more vulnerable to both stressors and leading to a more complete and long-lasting inhibitory effect against spoilage microorganisms.

In all cases, the antibacterial activity was greater against Gram-positive *S. aureus* than against Gram-negative *E. coli*. This difference may be due to the varying susceptibility caused by the respective cell wall structures [41]. This distinctive effect has also been observed by other authors in assays of antimicrobial agents in active packaging [42].

Several studies have investigated the addition of antimicrobial agents to plastic films in food packaging. Bibow et al. [43] and Suárez-Vega et al. [44] found that the presence of a hydroxyl group (−OH) attached to a carbon atom in the aromatic benzene ring in the structure of eugenol is crucial for its antimicrobial properties. On the other hand, packaging paper coated with olive extract rich in hydroxytyrosol has shown inhibitory effects on the growth of *Listeria monocytogenes* [34].

### 3.3. Evolution of pH and Water Activity in Stored Sponge Cake

The initial pH value on day 0 was 7.40, and it increased very slightly to an average value of 7.50 across all samples during the first 20 days of storage. In the SC-0 group under conventional air packaging, the pH value shifted toward alkalinity (pH 8.21) by day 30 of storage. In contrast, the pH in the rest of the groups remained stable with only slight variations around 7.40 until the end of storage. Therefore, the SC packaged in air showed a significant increase in pH values (*p* < 0.05), whereas storage in a modified atmosphere (40% carbon dioxide/60% nitrogen) kept the pH values essentially stable over the 70-day storage period.

The progressive pH increase observed in the control sponge cake (SC-0), which lacked active packaging protection, is primarily caused by microbial spoilage, specifically the metabolism of molds and yeasts that dominate the spoilage biota of medium-moisture baked goods. This increase is based on a combination of protein degradation, the production of volatile amines, and a buffering effect. As spoilage microorganisms (e.g., molds and spoilage bacteria) proliferate, they secrete enzymes (proteases) that break down the cake’s protein content (e.g., flour and egg proteins) into simpler compounds. This degradation process releases basic compounds, such as ammonia and various volatile amines. The accumulation of these basic compounds in the aqueous phase of the cake effectively raises the pH (i.e., reduces the concentration of H+ ions), causing the final pH to become more alkaline than the initial value.

The active packaging system minimizes this pH increase because the active compounds suppress the microbial activity responsible for protein breakdown. The combination of HOxTYR and EUG provides a synergistic antimicrobial effect that effectively delays or prevents the growth of the molds, yeasts, and bacteria that produce the alkaline amines. By controlling microbial proliferation, the active films stabilize the pH of the sponge cake over the 70-day storage period. Samples stored with the highly effective combined film (SC-4) showed a significantly lower or negligible pH increase compared to the control, confirming that the essential chemistry of the cake matrix was preserved and spoilage was successfully delayed.

Our results are consistent with those reported by Gonda et al. [45], who used MAP with 50% N_2_ and 50% CO_2_ for packaged SC. Similar results were also found by De La Rosa et al. [46], who reported alkaline mean pH levels (pH 7.40 to 8.76) throughout the experiment with sponge cakes stored for 120 days. It is well known that pH value significantly affects microbial growth and the speed of oxidation reactions, ultimately influencing food spoilage [47]. Most bacteria require an optimal pH level of around 7.0 to grow and multiply, whereas fungi require a broader range of pH levels. In contrast, Ardeshir et al. [48] reported a decreasing trend in pH of sponge cake during storage due to the presence of high amounts of polyphenols and organic acids, such as ascorbic acid.

The initial water activity (a_W_) value on day 0 was 0.79. During the first 20 days of storage, it decreased slightly to an average value of 0.77 for all samples. In the SC-0 group packaged in air, water activity decreased to 0.69 by day 30 of storage. In the other groups, however, it remained fairly stable with an average value of 0.76 until the end of storage. Therefore, SC packaged in air showed a decrease in water activity values, while storage in a modified atmosphere (40% carbon dioxide / 60% nitrogen) kept water activity values virtually unchanged during the 70-day storage period. For comparison, De La Rosa et al. [46] observed a continuous decreasing trend in water activity levels in sponge cake stored from initial levels between 0.77 and 0.80 to between 0.68 and 0.78 at 120 days.

In summary, packaging with MAP, with or without bioactive films, maintained stable pH values (around 7.40) and water activity (around 0.76) over the 70 days, unlike samples packaged in air (SC-0), which showed an increase in pH and a decrease in water activity, indicating faster deterioration.

### 3.4. Evolution of Height, Volume and Weight Losses and Texture in Stored Sponge Cake

**Figure 5:**
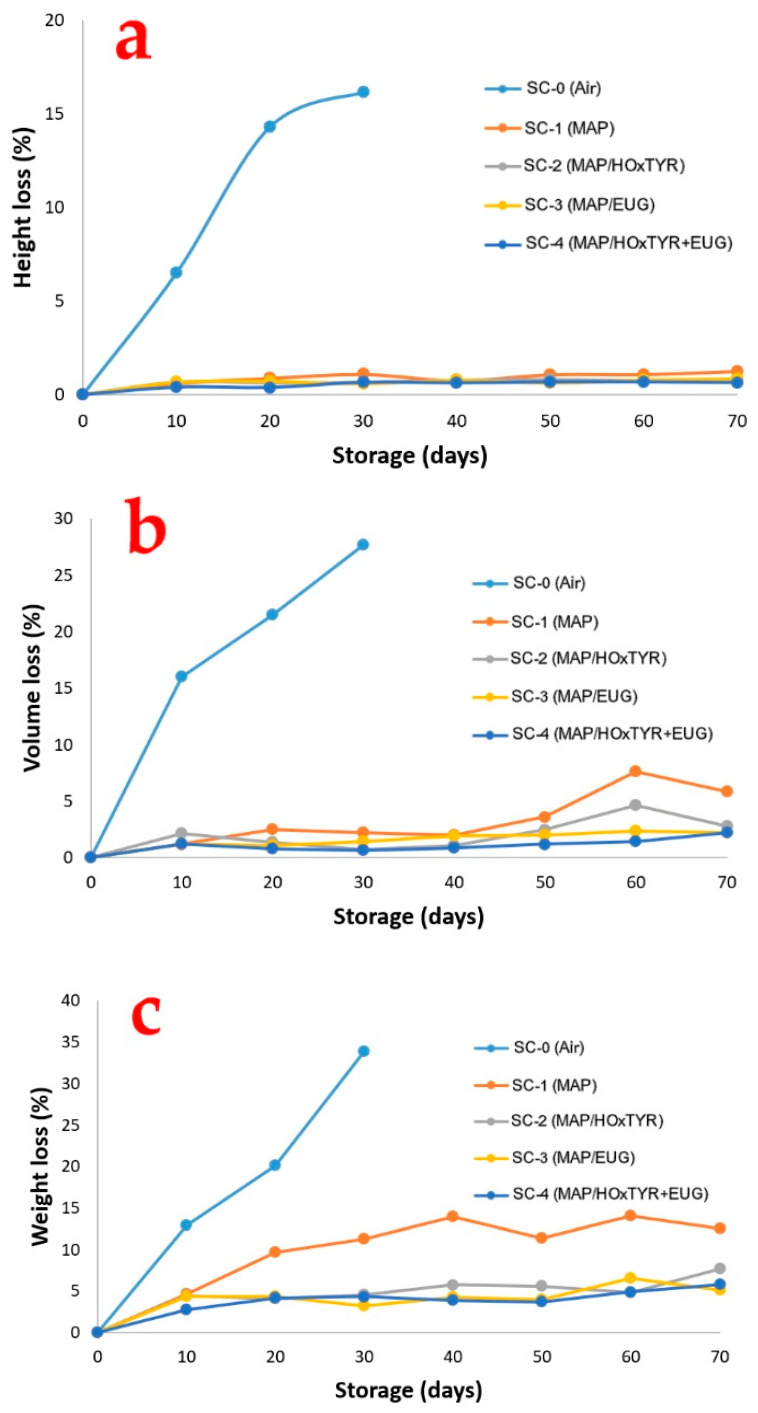
Losses in (**a**) height, (**b**) volume, and (**c**) weight of SC during storage at 15 °C for 70 days.

Our results showed that, during the first 30 days of storage, the loss of height in the SC-0 cakes (conventional air packaging) was significantly greater than in all other samples (Figure 5a), with the SC-4 group cakes showing the least loss (*p* < 0.05). Cakes packaged with PS film and stored in a modified atmosphere (MAP) consequently retained their volume better, maintaining better consumer appeal.

Under aerobic conditions (SC-0), the product sagged by 0.40, 0.86, and 1.04 cm after 10, 20, and 30 days of storage, respectively. This equates to height losses of 6.49%, 14.29%, and 16.13%. These observations are supported by the significant volume loss during storage, as shown in Figure 5b. The SC-0 group packaged in air experienced a 27.64% decrease in volume after 30 days of storage, corresponding to a loss of 49.69 cm^3^, indicating poor storage conditions. In contrast, SC samples packed under MAP showed minimal volume loss throughout the storage period, especially in groups SC-2, SC-3, and SC-4, whose films contained bioactive compounds. Again, the SC-4 group cakes exhibited the least volume loss (*p* < 0.05).

Regardless of the packaging type, the SC samples lost between 3.27 and 39.06 g (6.21% to 33.91%) of weight by the end of the storage period, compared to their initial weights (which ranged between 99.50 and 120 g). Weight losses were significantly greater in the SC-0 samples (air-packaged) compared to the other MAP-packaged samples (Figure 5c). Furthermore, SC-1 samples showed greater volume losses than those from groups SC-2, SC-3, and SC-4 (which contained bioactive compounds). The least loss of weight was likewise observed in the SC-4 group cakes (*p* < 0.05). The reduction in weight is primarily linked to moisture loss during storage. The use of N_2_ as an inert gas in MAP may restrain water migration and, consequently, minimize weight loss.

According to the texture profile analysis, the hardness (firmness) of the samples increased with storage time from the initial value of 372 g (day 0). The hardness of the control samples SC-0 (air-packaged) and SC-1 (MAP-packaged) increased rapidly, reaching 1000 g and 504 g on day 30, respectively. By the end of storage (day 70), samples from the SC-1 group (without bioactive agents) reached a hardness of 705 g.

However, the presence of bioactive films in groups SC-2, SC-3, and SC-4 significantly delayed the onset of hardening in the cakes, with final hardness values on day 70 of 571.51, 583.91, and 521.9 g, respectively. The combined application of HOxTYR and EUG (SC-4) had a more positive effect than the application of either compound separately. Overall, PS-based bioactive films had a great influence on maintaining the softness of the sponge cake samples during storage at room temperature.

A comparative analysis was performed between the observed weight loss (primarily driven by water evaporation/loss of volatiles) and the change in water activity (a_W_) over the storage period for all samples. In the SC-0 (control) and SC-3 samples, a positive correlation between the loss of weight/volume/height and the reduction in water activity was confirmed. This is expected, as moisture loss is the primary cause of product shrinkage and hardening (increased firmness) in baked goods.

The samples protected by the HOxTYR-containing films (SC-2 and SC-4) exhibited lower rates of weight loss, height reduction, and volume reduction. This physical stability directly correlates with their minimal change in water activity over the 70 days. This indicates that the incorporation of bioactive compounds (particularly the combination) resulted in better preservation of the cake structure, helping to retain intrinsic moisture better than the control.

The increase in cake firmness (staling) is accelerated by water loss, but it is also highly influenced by microbial metabolism. The samples with the lowest increase in pH (the active SC-4 film) also maintained the lowest increase in firmness. This confirms that the antimicrobial action (preventing protein/carbohydrate breakdown) contributed significantly to preserving the cake’s original texture and elasticity, going beyond mere water retention.

The most significant loss of structural integrity (volume/height) was observed in the SC-0 and SC-3 samples at the point where mold growth and microbial activity became evident. Microbial growth can lead to enzymatic degradation of the cake matrix, causing collapse and severe structural damage. The active SC-4 film prevented this deterioration, maintaining the initial structure until the end of the trial.

Thus, the firmness data show that the SC-4 films significantly inhibit the increase in hardness associated with staling and water loss. This is a critical finding, as consumer acceptance of sponge cake rapidly declines once the firmness increases significantly, typically when the measured initial firmness has increased by 50% to 100%. The superior textural stability demonstrated by our active packaging is a primary factor supporting its ability to meet consumer quality expectations for an extended shelf life.

In general, sponge cakes are considered more desirable to consumers if they have a large volume, as it indicates better aeration and a lighter, fluffier crumb [49]. However, this characteristic generally deteriorates over time due to physical-chemical changes that occur during storage. Karambakhsh et al. [50] reported that cake volume decreased with storage period due to moisture loss, lipid oxidation, and starch retrogradation, which are natural processes that occur over time, especially at room temperature. The role of MAP and active films on volume stability and textural quality of bakery products is well established [51,52]. These packaging methods help control factors like moisture loss, microbial growth, and oxidation, all of which impact a cake’s textural properties and overall quality. Other researchers have reported that applying essential oils to the active packaging of bakery products results in lower water content losses during storage, arguing that these bioactive compounds can act as a barrier against moisture loss due to their hydrophobic nature [53].

Moisture loss during storage is a decisive factor in explaining the hardening and aging of stored cakes [54]. Consequently, the combined use of MAP and bioactive films can effectively prevent some of the processes involved in the hardening and aging of stored cakes.

### 3.5. Evolution of Lipid Oxidation in Stored Sponge Cake

Our results demonstrated that the lipid oxidation index (TBARS), which measures secondary oxidation products like malondialdehyde (MDA), increased with storage time (Table 4). This trend is attributed to the gradual breakdown of triglycerides and the resulting production of free fatty acids. The initial lipid oxidation index (day 0) of all cakes was very low, measured at approximately 0.49 mg MDA/kg. Lipid oxidation values for the SC-0 samples (conventional air packaging) were not measured after 30 days of storage due to visible mold growth on the surface.

On the twentieth day, both the air-packaged samples (SC-0) and the MAP samples (SC-1) had high TBARS values, reaching 1.68 and 0.91 mg MDA/kg, respectively. The TBARS of the air-packaged samples then increased to 2.06 mg MDA/kg on day 30, a value well above the detection limit for a sensory panel. The MAP SC-1 samples maintained a relatively stable TBARS value around 1 mg MDA/kg until day 50 of storage. However, by the end of the storage period on day 70, the TBARS of these samples had increased to almost 2.0 mg MDA/kg, a value significantly higher than that of the SC-2 and SC-4 samples, which contained antioxidants.

The combined effect of HOxTYR and EUG incorporated into the SC-4 active film resulted in the lowest oxidation rates during storage. Specifically, between days 60 and 70, samples SC-2 (MAP/HOxTYR) and SC-3 (MAP/EUG) had high TBARS values (ranging between 1.31 and 1.94 mg MDA/kg, respectively), while SC-4 samples (MAP/HOxTYR + EUG) maintained significantly lower values (between 0.98 and 1.44 mg MDA/kg).

It can be concluded that the greater delay in lipid oxidation in the SC-4 samples is a direct result of the synergistic combination of hydroxytyrosol and eugenol, which also demonstrated the best performance in the in vitro DPPH test (IC_50_ = 29.43; AAI = 1.37). The presence of a hydroxyl group (-OH) attached to a carbon atom in the aromatic benzene ring in both HOxTYR and EUG is crucial for their potent antioxidant properties [34,43].

Although a study of the release kinetics of the active compounds was not conducted, the expected release behavior is inferred from their physicochemical properties. The highly volatile eugenol (EUG) is expected to exhibit an initial fast release into the headspace, while the more hydrophilic hydroxytyrosol (HOxTYR) should show a sustained effect on radical regeneration within the cake matrix.

Our lipid peroxidation results align with those obtained in related studies [55,56,57,58]. This study clearly demonstrates that enriching PS-based films with HOxTYR provides a significant antioxidant effect during storage. Given its recognized health properties and its ability to act synergistically with EUG in terms of antioxidant action, HOxTYR is a promising candidate for improving the preservation of baked goods and bakery products, serving as a replacement for synthetic additives.

### 3.6. Evolution of Crumb Color in Stored Sponge Cake

The color characteristics of the sponge cake (SC) crumb throughout storage are shown in Table 5. The CIELab color space was used, measuring three coordinates: L*, the lightness (from 0 to 100), as well as a*, the green-red component, and b*, the blue-yellow component. By measuring the color difference parameter ΔE* (Delta E), we were able to accurately assess color consistency and detect any deviation from the freshly baked state. The freshly manufactured SC (day 0) presented a bright yellow color, with initial L*, a*, and b* values of 70.61, 0.52, and 31.71, respectively. The L*, a*, and b* values of the SC crumb decreased slightly during storage, which is a common observation in baked goods.

In terms of ΔE*, the analysis of color parameters (Table 5) showed that samples SC-2 (MAP/HOxTYR) and SC-4 (MAP/HOxTYR + EUG) exhibited significantly greater color stability throughout the storage period compared to the other samples (*p* < 0.05). Their maximum ΔE* values were less than 2.50, remaining below the threshold detectable by the human eye (typically considered 3.00). This indicates that these samples showed no visually detectable differences compared to the freshly baked SC (day 0). Based on the ΔE* results, consumer panels would have been able to visually discriminate between the control samples (SC-0 Air and SC-1 MAP) and the other treated samples because their ΔE* values were higher than 3.00.

SC color changes during storage are influenced by several factors, including storage conditions and the presence of bioactive films with antioxidant and antimicrobial properties. SC color can also be affected by the addition of eggs [59] and other colored ingredients used in the formulation [60]. Furthermore, SC crust color is affected during baking when the temperature exceeds 100 °C and Maillard and caramelization reactions occur. Natural bioactive compounds, particularly hydroxytyrosol, played a significant role in the color stability of the SC during storage.

The SC-1 cakes packaged under MAP but without the active film maintained color stabilization for up to 50 days of storage (ΔE* = 2.81). In contrast, the SC-0 cakes, packaged under conventional air packaging, lost all color stability (ΔE*= 4.17) even by the tenth day of storage. After 30 days of storage, these SC-0 samples showed superficial fungal growth, so the color study was discontinued. Considering the influence of moisture content and water activity (a_W_) of the SC on product color, it was observed that samples with higher a_W_ values had greater luminosity (L*). SC-0 samples packaged under aerobic conditions had the lowest luminosity (L* = 40.46 to 68.50). However, in the SC-4 samples, which were packaged in MAP with the HOxTYR + EUG film, the ΔE* value decreased from day 10 to day 60, indicating that crumb color stabilization was successfully achieved. It was observed that the SC samples packaged with HOxTYR (SC-2) were more color-stable than the controls (SC-0 and SC-1) and the SC-3 samples packaged only with the EUG film. Already on the tenth day of storage, the ΔE* values of the SC-2 and SC-4 samples with bioactive compounds were 66.66–86.57% lower than those observed in the control samples SC-0 and SC-1. By the end of storage (70 days), the differences in ΔE* values between the control SC-1 and the SC-2 and SC-4 groups exceeded 80%.

Furthermore, during storage, the L* of the control samples decreased, which could be attributed to fat oxidation and evaporative water loss, expressed as weight loss. It appears that MAP alone, or in combination with an antioxidant bioactive film based on HOxTYR and EUG, can delay product degradation, thus preserving its color stability during storage. In fact, control SC-0 cakes packaged under aerobic conditions showed the lowest L* and b* values—59.97 and 23.06, respectively—on day 20 of storage, characteristic of a deteriorated and less attractive color. Likewise, these control SC-0 samples showed a b*/a* ratio value of 37.80 on the twentieth day of storage, which was significantly lower (*p* < 0.05) than the initial value of 60.98 (day 0). In contrast, samples SC-2 and SC-4 showed better color retention even at the end of storage (70 days), displaying a more vibrant color given their higher b*/a* ratio values of 51.03 and 45.86, respectively.

The main reactions contributing to the visible crumb color degradation during sponge cake storage are lipid oxidation and continued non-enzymatic browning (Maillard reaction). Lipid oxidation is the most significant factor over the storage time. The degradation of unsaturated fatty acids leads to the formation of volatile and non-volatile secondary oxidation products (e.g., aldehydes like malondialdehyde, which is measured by the TBARS assay, and ketones). These highly reactive products can undergo further non-enzymatic browning reactions by reacting with amino acids and proteins (similar to the final stages of the Maillard reaction), resulting in the undesirable yellowing and browning of the crumb. Furthermore, free radicals generated during lipid oxidation can cause the degradation or bleaching of natural pigments (e.g., carotenoids from egg yolk and fat). While most Maillard browning occurs during baking, the reaction continues at lower rates during storage, particularly as moisture migrates and reactive intermediates concentrate.

Regarding the role of MAP with low O_2_ and active compounds, the incorporation of potent antioxidants (HOxTYR and eugenol) synergistically stabilizes the color by interrupting the lipid oxidation chain reaction. The low oxygen atmosphere physically restricts the availability of the primary oxidant. Simultaneously, HOxTYR and EUG, being excellent free-radical scavengers, rapidly quench lipid peroxyl radicals. This dual action significantly reduces the formation of the chromophoric secondary oxidation products, thereby maintaining the crumb’s original L* (lightness) and hue, as demonstrated by the lower ΔE* and TBARS values in the combined treatment.

While the primary control of color change is due to antioxidant activity, pH stability is also a key quality parameter. Changes in pH (a decrease due to microbial production of organic acids or lipolysis, or an increase due to protein degradation/microbial spoilage) can affect the rate of the Maillard reaction intermediates and the stability and color of certain natural pigments. In this sense, the packaging treatments that best preserved color (low ΔE*) were also the most effective at stabilizing the pH and suppressing microbial growth. This demonstrates that the combined MAP and antioxidant strategy provides comprehensive quality preservation, where color stability serves as a strong visual indicator of suppressed oxidative spoilage and microbial activity.

The color of SC is a key quality factor, influenced by the recipe, the baking process, and storage. Numerous studies have been conducted on the role color plays in how we perceive and even appreciate food. Research shows that our perception of flavors is greatly influenced by food color. According to some experts, “we eat with our eyes before even smelling or tasting the product” [61]. An attractive color can encourage purchase, while a deteriorated color can discourage consumers [62,63]. Color variation in stored SC can be due to many factors already mentioned, such as moisture loss, oxidation, pH stability, and microbial spoilage. Therefore, it is necessary to analyze the origin of these variations in order to implement appropriate measures, such as improving packaging.

### 3.7. Evolution of Microbial Counts in Stored Sponge Cake

Table 6 shows the total viable counts obtained from cakes stored under different conditions over a 70-day period. From day 0 to day 10, no contamination (< 1 log CFU/g) was reported in any of the batches studied. Aerobically stored SC-0 samples already showed high total viable counts in PCA on the twentieth day, reaching a maximum of 7.30 log CFU/g after 30 days of storage, which is above the acceptance limit set at 6 log CFU/g.

The use of Modified Atmosphere Packaging (MAP) significantly slowed microbial growth over the 70 days of storage. However, microbial counts increased in all MAP samples, albeit slowly, despite the presence of a CO_2_-rich atmosphere. This growth was likely enabled by residual O_2_ occluded in the large number of pores within the cake matrix, even though the O_2_ in the headspace of all packaged samples remained extremely low (around 0.01%).

Furthermore, the synergistic application of MAP and active packaging systems showed great potential to delay microbial growth. At the end of storage, all SC samples packaged with active systems (SC-2, SC-3, and SC-4) showed satisfactory microbiological quality according to established criteria [64,65]. According to the results obtained, the antimicrobial effect was greater in SC-3 (active film with eugenol) than in SC-2 (active film with hydroxytyrosol) and was maintained in SC-4 (active film with the combination of HOxTYR and EUG).

In recent years, other studies have indicated the antimicrobial capacity of hydroxytyrosol [20] and eugenol [66] as natural bioactive agents in a variety of packaged foods. Polymeric films containing eugenol have recently attracted increasing interest in the food industry [67,68]. Furthermore, they have been successfully used to control bacterial biofilm growth on stainless steel surfaces [69,70].

Table 6 also shows the total mold counts obtained from cakes stored under different conditions over the 70-day period. From day 0 to day 10, no contamination was reported in any of the batches studied (< 1 log CFU/g). Our results show that at 70 days of storage, mold counts were lower than 2 log CFU/g in the MAP samples, compared to counts of 4.30 log CFU/g found in the SC-0 samples under conventional air packaging, which showed surface molding at 30 days of storage (the limit of acceptability of yeasts and molds was set at 3 log CFU/g). Up to day 60 of storage at room temperature, no evidence of mold was detected in samples SC-3 and SC-4, which contained eugenol in the active film, representing a significant difference compared to the other groups (*p* < 0.05). Of the film-activated groups, SC-4, consisting of a mixture of hydroxytyrosol and eugenol, exhibited the lowest mold counts.

Therefore, the onset of food spoilage in the air-packaged samples occurred around day 20 of storage. Our results are consistent with those reported by Gonda et al. [45], who used MAP (N_2_/CO_2_ 50/50) for cake packaging; mold growth was evident after 15 days in air-packaged cakes, whereas when elevated levels of CO_2_ were used in modified atmosphere packaging (MAP), mold growth was inhibited at 15 °C for at least 70 days. For comparison, Ben Miri et al. [21] demonstrated the efficacy of eugenol fumigation in reducing the mold *Aspergillus parasiticus* on green coffee beans stored for 12 months. They also verified the antifungal efficacy of cereal fumigation with menthol, eugenol, and a combination of both against *Aspergillus ochraceus* and *A. niger* [71]. The binary mixture of menthol/eugenol (1:1) exhibited synergistic effects against both fungi.

The surface of bakery goods is almost sterilized during the baking process, when mold spores are inactivated. However, additional cutting, contact with equipment and surfaces, and packaging procedures can be sources of fungal contamination [72,73]. The appearance of mold on the surface of bakery goods is a determining factor in the end of their shelf life, requiring their recall. Mold often forms as a surface layer on the crust or exposed parts of the product. The most common species belong to the genera *Aspergillus*, *Penicillium*, and *Rhizopus*. For food safety reasons, it is important to detect the presence of mold before signs of biodeterioration are observed, as some species produce mycotoxins [74].

Our study highlights the long-term efficacy of the combination of modified atmospheres and active films in preserving sponge cake over a 70-day period. The presence of a hydroxyl group (-OH) in the structure of HOxTYR and EUG is crucial for their antimicrobial properties [75]. This group is essential for both molecules to interact with cell membranes and disrupt fungal growth, ultimately leading to cell death. In our experimental design, there was no direct contact between the bioactive compounds and the cake. In fact, the protective effect of our active packaging system utilizes two different mechanisms, dictated by the physicochemical properties of the incorporated compounds: volatilization into the headspace for lipophilic eugenol and slow migration from the active film for hydrophilic hydroxytyrosol. Therefore, it can be stated that both natural compounds released from the active films were primarily responsible for inhibiting the growth of spoilage microorganisms.

The long study period (70 days) has provided valuable knowledge to determine the viability and long-term efficacy of the films for use in food packaging. The significant results observed in the quality parameters, such as lower lipid oxidation (TBARS) and reduced microbial growth in the MAP with HOxTYR + EUG film, serve as strong indirect evidence that both compounds were successfully released from the active films and effectively exerted their biological function. While the efficacy was demonstrated, the precise quantification of released compounds in the headspace was not performed. Therefore, a comprehensive study on the release kinetics and partitioning of hydroxytyrosol and eugenol between the film, headspace, and food matrix—using techniques like GC-MS or headspace-HPLC—is a necessary next step for system optimization and will be the subject of dedicated follow-up research.

The use of modified atmosphere packaging appears to be a promising technology for bakery products with intermediate moisture and neutral pH stored at ambient temperatures, as the main spoilage-causing microorganisms are aerobic. The strategic gases used in modified atmosphere packaging (MAP) for retail are carbon dioxide (CO_2_) and nitrogen (N_2_), with very low O_2_ levels (<1%). These very low O_2_ levels, combined with the bacteriostatic and fungistatic activity of CO_2_, prevent the development of microorganisms on the product surface [76]. It has been reported that at sufficiently high concentrations, CO_2_ acts as an effective preservative by inhibiting microbial growth and maintaining food freshness, thereby extending shelf life [77]. One contributing factor is thought to be the formation of carbonic acid on the surface of products, which negatively affects the growth of surface fungi [78]. However, CO_2_ levels above 70% are not recommended for bakery products [79].

In this study, residual CO_2_ and O_2_ in the headspace were monitored during cake storage. The initial gas composition (40% CO_2_, 0.21% residual O_2_, and the remainder N_2_ as inert gas) changed slightly during storage in all groups. In particular, CO_2_ concentrations decreased by approximately 7% during the first 30 days in all groups. The presence of active molecules in groups SC-2, SC-3, and SC-4 did not significantly affect CO_2_ or N_2_ in the headspace. In parallel, residual O_2_ concentrations also decreased in all groups to approximately 0.01%. This residual O_2_ value in the headspace is the critical value indicated by Piergiovanni and Fava [80] for the storage of perishable foods. The gradual decline in O_2_ content may be related to O_2_ consumption due to microbial activity. These results on the dynamics of headspace gas composition during cake storage are consistent with those of Janjarasskul et al. [51] in the study of shelf-life extension of sponge cake by active packaging.

### 3.8. Overall Acceptability and Shelf Life of Stored Sponge Cake

Sensory analysis plays a crucial role in a product’s commercial success and consumer satisfaction. Consumers often judge the quality and freshness of foods based on their sensory attributes. Therefore, the overall acceptability of the sponge cake was assessed in all groups throughout storage, using a panel of self-identified sponge cake consumers.

Starting with an initial acceptability of 6.25 points (on a scale where 5.0 is the “like slightly” threshold), the SC-0 samples under conventional air packaging showed a sharp decline in sensory quality. They reached a clearly unacceptable score of 1.30 points after only 30 days of storage (Table 7).

The remaining MAP-packaged groups consistently maintained overall acceptability scores above 5 points until 60 days of storage. From day 60 onwards, acceptability scores fell slightly below 5 points in the SC-2 (MAP/HOxTYR) and SC-3 (MAP/EUG) groups. However, acceptability remained above 5 points in both the SC-1 (MAP only) and SC-4 (MAP/HOxTYR + EUG) groups.

The bioactive film that best maintained the sponge cake’s sensory quality was the one containing the synergistic combination of hydroxytyrosol and eugenol (SC-4). Not surprisingly, this group also exhibited the lowest weight loss, the lowest hardness values, excellent freshness in terms of color and odor (low TBARS value), and the lowest microbial count. With an acceptability score of 5.20 points, these SC-4 samples fell into the “like slightly” quality category after 70 days of storage.

Furthermore, the panelists noted that no EUG or HOxTYR aromas were detected during the sensory evaluation. This is a key technical and commercial success for the active packaging system, as it demonstrates that the release of the bioactive compounds is controlled and effective in preservation without negatively impacting the cake’s characteristic aroma and flavor.

Our study measured the following key quality attributes: texture, odor/flavor, appearance, and shelf life. Instrumentally, the active packaging resulted in a lower increase in hardness compared to control samples. This was perceived as a smoother, less stale texture, confirming that maintaining texture directly contributes to a positive eating experience. Regarding odor, significantly lower TBARS values (indicating less lipid oxidation) led to the absence of rancid or off-flavors, thereby maintaining freshness. Low TBARS values are critical, as they ensure the flavor profile remains uncompromised by oxidation. In terms of appearance, the active packaging produced more stable color (ΔE*) and greater volume retention. This made the cake visually more appealing and attractive to the consumer, as reduced discoloration and collapse maintain consumer confidence. Finally, lower microbial counts explained the extended shelf life: the overall acceptability score remained above the critical acceptability threshold (“like slightly, ≥5.0) until day 70, at which point the cake was still appealing visually, texturally, and aromatically. In summary, the preserved quality parameters, when combined, were the driving force behind the final, positive consumer acceptance.

The overall acceptability of sponge cake deteriorates over time due primarily to aging, a process involving starch degradation and moisture redistribution, which results in a loss of softness and freshness. When packaged aerobically at room temperature (SC-0), the sponge cake maintains its acceptability for only a short period of a few days. However, modified atmosphere storage combined with bioactive packaging (SC-4) can extend shelf life to approximately 70 days.

In conclusion, the results of the sensory evaluation suggest that the use of the active film with HOxTYR and EUG would not compromise consumer acceptance for up to 70 days of storage at 15 °C.

## 4. Conclusions

The application of polystyrene (PS) films enriched with the natural bioactive compounds hydroxytyrosol (HOxTYR) and eugenol (EUG) represents a promising and relatively innovative approach for preserving bakery products. These natural compounds offer an environmentally friendly alternative to synthetic preservatives, which aligns with the growing demand for clean-label, sustainable food packaging solutions. Our study definitively demonstrated the efficacy of this system: the combination of HOxTYR and EUG in the active PS films exhibited a significantly greater synergistic antioxidant capacity compared to the individual molecules, and furthermore, the combined film showed a synergistic antibacterial effect against *S. aureus* and *E. coli*. When combined with modified atmosphere packaging (MAP), the new active film provided comprehensive, long-term protection against physicochemical, microbial, and sensory deterioration during the entire 70-day storage period at 15 °C. This optimal packaging system (MAP with HOxTYR + EUG film) successfully extended the sponge cake’s shelf life to 10 weeks (70 days) while maintaining satisfactory microbiological quality and low lipid oxidation. Crucially, sensory evaluation scores remained high, indicating that the active PS-based films are suitable for commercial use in terms of overall acceptability, introducing no undesirable off-flavors.

The proposed active packaging system holds significant promise for industrial applications, as its compatibility with existing, high-speed industrial packaging lines for bakery products means the film can be seamlessly integrated without requiring major infrastructure changes. However, several critical challenges must be addressed prior to large-scale adoption. Achieving cost-competitiveness relative to conventional synthetic antioxidants requires optimizing the active compound loading to ensure maximum efficacy at minimal dosage. Additionally, the manufacturing process for PS films involves elevated temperatures, which poses a risk of thermal degradation of the natural phenolics or loss of the volatile eugenol, necessitating careful process control to preserve compound integrity. Rigorous testing and regulatory clearance (e.g., FDA/EFSA approval) are also required for the specific use of this combined system in food packaging, particularly concerning non-migration limits and food contact safety.

Regarding the sustainability trade-off, it is acknowledged that PS presents environmental challenges due to its limited recycling infrastructure and non-biodegradability. However, we argue that the primary environmental benefit of this packaging system is derived from preventing food loss. This argument is based on the generally accepted principle that food waste often carries a greater environmental burden (e.g., land use, water, energy, and greenhouse gas emissions from production and disposal) than the packaging required to protect it. By extending the sponge cake’s shelf life until 70 days, the active PS film offers a net positive environmental impact by minimizing the wastage of resources invested in food production. To address material limitations for a fully sustainable solution, the proven efficacy of the HOxTYR + EUG combination can serve as a scientific basis for its integration into biodegradable matrices. Future research could focus on transitioning these active compounds to environmentally friendly polymers like polylactic acid (PLA), polyhydroxyalkanoates (PHAs), or starch- and cellulose-based films, with the aim of developing a fully sustainable and highly effective active packaging solution. As an immediate interim solution, using recycled polystyrene (rPS) as the base polymer for the active film is suggested to reduce environmental impact while maintaining the proven efficacy and lower cost of PS. Based on these promising results, we outline clear paths for subsequent work, including conducting migration kinetics studies to model the diffusion coefficients of EUG and HOxTYR, exploring the use of nano-encapsulation to further stabilize the active compounds for more controlled release and potential cost reduction, and testing the active packaging system on a wider range of bakery products (e.g., muffins, cookies, breads) to confirm its versatility across different food matrices.

## Figures and Tables

**Table 1:** Sliced cake sample group codes.

Sample Code	Sponge Cake Under Conventional Air Packaging
SC-0	Sponge cake under conventional air packaging
SC-1	Sponge cake packaged under modified atmosphere MAP (40% CO_2_ + 60% N_2_) with PS foamed film (PS) without bioactive compounds
SC-2	Sponge cake packaged under MAP with PS containing 1% hydroxytyrosol as bioactive compound
SC-3	Sponge cake packaged under MAP with PS containing 1% eugenol as bioactive compound
SC-4	Sponge cake packaged under MAP with PS containing 0.6% hydroxytyrosol plus 0.6% eugenol as bioactive compounds

**Table 2:** Antioxidant properties of the bioactive PS films measured by DPPH scavenging assay.

Parameter	PS/HOxTYR	PS/EUG	PS/HOxTYR + EUG
IC_50_ (µg/mL)	49.25 ± 0.09	69.28 ± 0.12	29.43 ± 0.21
Antioxidant activity index (AAI)	0.81 ± 0.02	0.58 ± 0.01	1.37 ± 0.02
Antioxidant activity	Moderate	Poor	Strong

**Table 3:** In vitro antimicrobial activity of PS-based films against *S. aureus* and *E. coli*. Results expressed in log CFU/mL and levels of inhibition in percentage (in brackets).

Food Pathogen	Control	PS/HOxTYR	PS/EUG	PS/HOxTYR + EUG
*S. aureus*	5.0	4.6 (8.0%)	2.9 (42.0%)	1.9 (62.0%)
*E. coli* O157:H7	5.0	4.8 (4.0%)	3.2 (36.0%)	2.1 (58.0%)

**Table 4:** Evolution of lipid oxidation (lipid peroxidation index TBARS) in SC during storage.

Sample Groups	Storage Period (Days)							
	0	10	20	30	40	50	60	70
SC-0 (Air)	0.49	0.86	1.68	2.06	na ^1^	na	na	na
SC-1 (MAP)	0.49	0.47	0.91	0.89	1.02	0.98	1.53	1.98
SC-2 (MAP/HOxTYR)	0.49	0.49	0.48	0.55	0.80	1.00	1.31	1.52
SC-3 (MAP/EUG)	0.49	0.48	0.51	0.81	0.97	0.96	1.51	1.94
SC-4 (MAP/HOxTYR + EUG)	0.49	0.49	0.43	0.53	0.75	0.85	0.98	1.44

^1^ not analyzed.

**Table 5:** Evolution of crumb color coordinates (L *, a *, b *) and total color difference (ΔE *) in SC during storage. In bold ΔE * values above 3.00, meaning the color difference is evident to the human eye.

CIELAB System	Storage Period (Days)							
	0	10	20	30	40	50	60	70
SC-0 (Air)								
L*	70.61	68.50	59.97	40.46	na ^1^	na	na	na
a*	0.52	0.31	0.61	0.81	na	na	na	na
b*	31.71	27.81	23.06	26.24	na	na	na	na
ΔE*	-	**4.17**	**13.36**	**30.26**	na	na	na	na
SC-1 (MAP)								
L*	70.61	70.74	70.68	70.03	69.35	67.63	66.51	65.16
a*	0.52	0.55	0.49	0.61	0.41	0.49	0.47	0.62
b*	31.71	30.74	30.11	28.95	29.05	31.37	31.00	30.54
ΔE*	-	1.11	1.61	2.79	2.73	2.81	**3.92**	**5.44**
SC-2 (MAP/HOxTYR)								
L*	70.61	70.26	70.83	70.87	70.04	69.93	68.67	67.86
a*	0.52	0.51	0.40	0.35	0.40	0.50	0.52	0.61
b*	31.71	31.10	31.33	30.86	30.38	30.88	31.01	31.13
ΔE*	-	0.56	1.11	1.02	1.23	1.03	1.70	2.46
SC-3 (MAP/EUG)								
L*	70.61	69.20	69.00	68.77	69.15	68.23	67.14	66.89
a*	0.52	0.55	0.52	0.71	0.70	0.51	0.62	0.53
b*	31.71	30.73	30.97	31.43	29.23	30.32	30.18	30.20
ΔE*	-	1.39	1.60	1.90	2.76	2.51	**3.46**	**3.65**
SC-4 (MAP/HOxTYR + EUG)								
L*	70.61	70.54	70.60	70.48	70.50	69.65	70.34	68.64
a*	0.52	0.53	0.49	0.60	0.39	0.51	0.55	0.67
b*	31.71	31.73	30.96	31.08	30.96	31.74	31.34	30.73
ΔE*	-	0.99	0.83	0.75	0.80	0.87	0.71	1.87

^1^ not analyzed.

**Table 6:** Evolution of total viable count and mold count in SC during storage.

Days	Total Viable Count in PCA (log CFU/g)					Mold Count in PDAC (log CFU/g)				
	SC-0	SC-1	SC-2	SC-3	SC-4	SC-0	SC-1	SC-2	SC-3	SC-4
0	<1	<1	<1	<1	<1	<1	<1	<1	<1	<1
10	2.20	<1	<1	<1	<1	2.77	<1	<1	<1	<1
20	5.30	1.30	1.18	<1	<1	3.20	<1	<1	<1	<1
30	7.30	2.20	2.35	1.18	<1	4.30	<1	<1	<1	<1
40	na ^1^	2.77	2.56	1.60	1.18	na	<1	<1	<1	<1
50	na	3.01	2.86	1.70	1.30	na	1.18	1.18	<1	<1
60	na	3.18	3.11	1.85	1.40	na	1.65	1.48	<1	<1
70	na	3.68	3.65	2.10	2.10	na	1.70	1.65	1.18	1.00

^1^ not analyzed.

**Table 7:** Evolution of overall acceptability in SC during storage in a 7-point hedonic scale (7 = like extremely, 6 = like moderately, 5 = like slightly, 4 = neither like nor dislike, 3 = dislike slightly, 2 = dislike moderately, and 1 = dislike extremely).

Sample Groups	Storage Period (Days)							
	0	10	20	30	40	50	60	70
SC-0 (Air)	6.25	4.05	1.85	1.30	na ^1^	na	na	na
SC-1 (MAP)	6.25	6.05	6.15	5.85	6.00	5.70	5.45	5.10
SC-2 (MAP/HOxTYR)	6.25	6.30	6.15	5.95	5.60	5.35	5.10	4.90
SC-3 (MAP/EUG)	6.25	6.30	6.30	6.05	5.75	5.45	5.00	4.75
SC-4 (MAP/HOxTYR + EUG)	6.25	6.60	6.50	6.28	5.95	5.75	5.40	5.20

^1^ not analyzed.

## Data Availability

Data is contained within the article.
